# Properties enhancement of carboxymethyl cellulose with thermo-responsive polymer as solid polymer electrolyte for zinc ion battery

**DOI:** 10.1038/s41598-020-69521-x

**Published:** 2020-07-28

**Authors:** Isala Dueramae, Manunya Okhawilai, Pornnapa Kasemsiri, Hiroshi Uyama, Rio Kita

**Affiliations:** 1 Metallurgy and Materials Science Research Institute, Chulalongkorn University, Bangkok, 10330 Thailand; 2 Center of Excellence in Responsive Wearable Materials, Chulalongkorn University, Bangkok, 10330 Thailand; 3 Sustainable Infrastructure Research and Development Center and Department of Chemical Engineering, Faculty of Engineering, Khon Kaen University, Khon Kaen, 40002 Thailand; 4 Department of Applied Chemistry, Graduate School of Engineering, Osaka University, Suita, Osaka 565-0871 Japan; 5 Department of Physics, Tokai University, Kanagawa, 259-1292 Japan

**Keywords:** Batteries, Energy, Batteries, Electrochemistry

## Abstract

A novel polymer host from carboxymethyl cellulose (CMC)/poly(*N*-isopropylacrylamide) (PNiPAM) was developed for a high safety solid polymer electrolyte (SPE) in a zinc ion battery. Effects of the PNiPAM loading level in the range of 0–40% by weight ( wt%) on the chemical, mechanical, thermal, and morphological properties of the CMC/PNiPAMx films (where x is the wt% of PNiPAM) were symmetrically investigated. The obtained CMC/PNiPAMx films showed a high compatibility between the polymers. The CMC/PNiPAM20 blend showed the greatest tensile strength and modulus at 37.9 MPa and 2.1 GPa, respectively. Moreover, the thermal degradation of CMC was retarded by the addition of PNiPAM. Scanning electron microscopy images of CMC/PNiPAM20 revealed a porous structure that likely supported Zn^2+^ movement in the SPEs containing zinc triflate, resulting in the high Zn^2+^ ion transference number (0.56) and ionic conductivity (1.68 × 10^–4^ S cm^−1^). Interestingly, the presence of PNiPAM in the CMC/PNiPAMx blends showed a greater stability during charge–discharge cyclic tests, indicating the ability of PNiPAM to suppress dendrite formation from causing a short circuit. The developed CMC/PNiPAM20 based SPE is a promising material for high ionic conductivity and stability in a Zn ion battery.

## Introduction

With the environmental concerns and the limitation of fossil fuels as well as the growing energy demands, the rechargeable battery is one kind of renewable energy sources which is consecutively developed for a sustainable energy supply. In spite of the most promising technology of lithium-ion batteries (LIBs), the zinc ion battery is used as a compelling alternative battery chemistry to LIBs for energy storage system^1–3^ owing to its low cost, abundance, inflammability, low toxicity, promising energy density and environmental friendlier^4,5^. The utilization of metal-ion batteries as power sources has led to exhaustive research on electrolyte systems with high electrochemical performance. It plays a significant role in battery electrochemistry that is the medium for the movement of ions within the batteries.

Recently, solid polymer electrolytes (SPEs) have interested raised attention and enhanced research efforts as a fascinating alternative electrolyte bridge to liquid polymer electrolyte (LPEs) due to a lot of benefits, i.e., high durability, high energy density, lightweight, great flexibility for cell design, inert towards the electrodes, overcome the limitation of solvent leakage and low volatility, reduce the cell assembly cost, and great electrochemical and thermal stability^6^. Fundamentally, SPEs consist of a dissolution of metal salts, e. g. lithium and zinc salt, in a host polymer, which the hopping process is used to describe the metal ions movement along the amorphous phase of polymer after the salts dissociate by interaction with the polar group of polymer^7^. To coordinate with ions, therefore, the host polymer must contain electron donor groups such as O, N, and S. Moreover, it should provide fast segmental motion characteristic of polymer chains with low glass transition temperature, high degradation temperature, and spatial conformation to dissociate more salt concentration^8^. A lot of research has been performed to develop SPEs to become viable for use in electrochemical energy storage devices^1,9–15^. Synthetic polymers, such as polyethylene oxide (PEO)^1,9^, polyethylene glycol-based waterborne polyurethane^10^, polyacrylonitrile^11–13^, and polyvinyl alcohol (PVA)^13–15^, have been increasingly utilized as the main host polymers, but these polymers are costly, deplete petroleum resources, and trigger environmental problems, compared to natural polymers. Recently, the use of biopolymer materials has raised special attention as they are abundant in nature and are eco-friendlier. Several polymers have been found to be suitable as a polymer host, including chitosan^16,17^, cellulose^2,18,19^, agarose^20^, and carboxymethyl cellulose (CMC)^21–26^. Of these, CMC shows a good potential to act as a polymer host for proton-conducting biopolymer electrolytes.

The structure of CMC is a linear polymeric derivative of cellulose that consists of *β*-linked glucopyranose residues with partial hydroxyl groups substituted with carboxymethyl (–CH_2_COO–) groups. It is a biodegradable, low production cost, low environmental toxicity, and semi-crystalline material that exhibits an excellent film forming ability^21^. Moreover, CMC is an anionic polymer that can establish a strong linkage with oppositely charged materials, forming a polyelectrolyte complex^27^. The use of CMC as a single host polymer for SPEs has been reported^22,26,27^. For instance, the porous structure of a CMC-based gel polymer electrolyte (GPE) was fine-adjusted by varying the composition of *N, N*-dimethyl formamide, and used as a host for the lithium hexaflourophosphate electrolyte uptake. The ionic conductivity and ionic lithium transference of the optimum porosity GPE were reported at room temperature to be 0.48 mS cm^−1^ and 0.46, respectively. However, the tensile strength was significantly reduced (approximately 38%) from the original CMC^26^.

The enhancement of polymer properties through polymer blending or copolymers^13,16,17,23–25,28^, plasticization^22,29^ and composite formation^3,12^ have been competitively considered in the research community. The polymer blend technique was applied in the present study due to its low cost and easy processing as well as its ability to provide promising conductivity values. Moreover, this method is robust, and the desired properties can be tailored^16,17,23–25,28^.

Indeed, CMC has been blended with different polymers to form SPEs, and has achieved an ionic conductivity in the range of 10^−7^ to 10^−4^ S cm^−1^. For instance, the incorporation of CMC into carrageenan allowed the manipulation of the polymer structure and increased the conductivity via the formation of hydrogen (H)-bonds^18^. The CMC/PVA-based hybrid polymer system with different CMC: PVA molar ratios has been prepared via solution casting, where the 4:1 molar ratio CMC: PVA was found to be optimal and gave an increased conductivity by one order of magnitude from 10^−7^ to 10^−6^ S cm^−1^ compared to CMC, and showed the lowest level of crystallinity^25^. However, both SPEs systems have not reported the mechanical properties, which are insufficient for practical applications, and also critical intrinsic factors that influence the safety in use and large-scale manufacture of lithium batteries^2,16,18,23^. We present the bending of CMC with synthetic polymer. They could combine the characteristic properties of synthetic polymers like good mechanical and thermal properties and chemical stability with high proton-conducting of CMC due to the swelling ability of the –OH functional groups.

Thermo-responsive polymers based synthetic polymers are defined as those that are capable of conformational and chemical changes in response to environmental factors, such as temperature, pH, humidity, light, specific ions or molecules, electrical fields, solvent and ionic strength, etc. Poly(*N*-isopropylacrylamide) (PNiPAM) is a well-known thermo-responsive polymer, the properties of which can be fine-tuned at 32 °C^30^, a temperature close to that of the human body, via the switchable hydrophilic amide group (–CONH–) and hydrophobic propyl [–CH(CH_3_)_2_] moieties in the monomer structure. These polymers have been established to provide an efficient functionality of thermal self-protection, by preventing thermal runaway in lithium-metal batteries by efficiently inhibiting the ionic or electron conduction between electrodes beyond an unsafe temperature^31^.

Therefore, the recent attention to SPEs has focused on achieving high ionic conductivities as well as good mechanical properties and the ability for long term utilization with a high degree of safety. In this work, polymer blended SPEs were fabricated by the incorporation of different amounts of the thermo-responsive PNiPAM in CMC, and their physical, mechanical, and thermal properties, ionic conductivity, and electrochemical properties were characterized. The effect of the addition of zinc triflate (Zn(CF_3_SO_2_)_2_, or Zn(Tf)_2_, on the ion conductivity characteristics in SPEs was investigated.

## Materials and sample preparation

### Materials

Sodium CMC was purchased from the Changshu Wealthy Science And Technology Co., Ltd., China (White powder, viscosity of 2,580 mPa s for 2 wt% solution at 25 °C, degree of substitution = 0.76). The NiPAM monomer (> 99%) and all solvents (acetone, benzene, and hexane) were purchased from Wako Pure Chemical Industries, Ltd. The NiPAM was recrystallized from a 1:1 (v/v) ratio of benzene: hexane before use. 2,2′-Azo-bis(isobutyronitrile) (AIBN), used as initiator of polymerization, was purchased from Tokyo Chemical Industry Co., Ltd., while Zn(Tf)_2_ was purchased from Sigma-Aldrich Corporation.

### Polymerization of NiPAM to PNiPAM

The PNiPAM was synthesized by free radical polymerization. A 10:1 mol ratio of NiPAM (15.180 g) and AIBN (2.185 g) was dissolved in benzene (120 mL) in a 250-mL two-neck reactor fitted with an inlet and outlet for nitrogen (N_2_) bubbling, and a reflux condenser. The reaction mixture was stirred with a magnetic stir bar and kept at 56 °C for 90 min. The solvent was then evaporated from the product in a rotary evaporator and precipitated using acetone and hexane as a solvent and co-solvent, respectively. The obtained PNiPAM solution (in acetone) was fractionated several times by phase separation in a liter of n-hexane. The weight averaged molecular weight (*M*_w_) of all fractionated PNiPAM is in the range of 3.30 × 10^3^ to 78 × 10^3^ g mol^−1^. The fraction used in this study had a *M*_w_ of 3.30 × 10^3^ g mol^−1^ with a polydispersity (*M*_w_/*M*_n_) of 2.34, as characterized by gel permeation chromatography (GPC; TOSOH HLC-8220GPC) at 30 °C on TSK gel Super Multipore HZ-M column a with a retention index detector. Poly(ethylene glycol) and PEO were used as standards. The standard and samples were dissolved in 0.1 M NaCl at a concentration of 1.0 g L^−1^ and the GPC was eluted at a mobile phase flow rate of 1.0 mL min^−1^. We designed the blending of CMC with the lowest *M*_w_ of PNiPAM due to the expecting of plasticizing effect, which could enhance the ionic conductivity of SPEs with remaining of the mechanical properties. Moreover, the obtained PNiPAM provided a high amount of lowest *M*_w_. The *M*_w_ dependency of PNiPAM on the ionic conductivity of SPEs will be studied in the future of our research group.

### Preparation of the SPEs

The CMC/PNiPAMx-based SPEs (where x is the wt% of PNiPAM) were fabricated by solution casting. The CMC was dissolved in deionized water to a solid content of 1 wt%. Then PNiPAM was added and dissolved in the CMC solution to the desired weight ratio (0–50 wt%). To obtain the CMC/PNiPAMx mixed polymer with excellent properties, Zn(Tf)_2_ was added and dissolved to a weight ratio of 0–30 wt%. Each respective solution was then poured into an aluminium mould and dried at room temperature for 3 days. The composition of the different polymers before addition of Zn(Tf)_2_ is reported in Table 1.

**Table 1:** Thermal degradation parameters and ionic conductivity of CMC with different PNiPAM content.

Name	PNiPAM content ( wt%)	Char yield at 500 °C (%)	Initial temperature of main degradation (°C)	Ionic conductivity 10^−4^ S cm^−1^
CMC	0	39.3	185	1.22
CMC/PNiPAM10	10	34.5	250	0.57
CMC/PNiPAM20	20	37.3	255	1.68
CMC/PNiPAM30	30	50.3	259	0.56
CMC/PNiPAM40	40	46.7	266	0.66
PNiPAM	100	25.9	320	–

## Characterization methods

### Fourier-transform infrared spectroscopy (FT-IR)

The chemical structure of the pure polymers and blends was characterized by FT-IR using a Horiba FT-IR 720 spectrometer equipped with an attenuated total reflectance accessory. All obtained spectra were averaged from 64 scans at a resolution of 4 cm^−1^ within the spectral range of 650–4,000 cm^−1^. Background measurements were performed and subtracted from the readings.

### Measurement of the mechanical properties

To investigate the effect of the PNiPAM content in the CMC/PNiPAM blend on their tensile strength and tensile modulus at room temperature, a universal testing machine (Introns Co., Ltd model 5567) was used. The SPE films were prepared at 8 mm × 80 mm × 4 mm (length × width × depth). The test method was a tensile mode with a supporting span of 4 mm, using a crosshead speed of 4 mm min^−1^.

### Measurement of the thermal properties

Thermal properties, such as the glass transition temperature, melting temperature, and crystallization temperature, were measured using differential scanning calorimetry (DSC; METTLER STARe model). The SPEs samples were sealed in the aluminium pan and analysed at a heating rate of 10 °C min^−1^ from 30 to 280 °C under a N_2_ flow of 50 mL min^−1^. An empty pan was used as the reference.

The thermal stability of the SPE samples was characterized by thermogravimetric analysis (TGA; Mettle Toledo TGA–DSC). A sample weight of ~ 7 mg was contained in aluminium oxide and operated from 30 to 550 °C at a heating rate of 20 °C min^−1^.

### Scanning electron microscopy (SEM)

The morphology of the dried samples was evaluated using SEM analysis with a Hitachi SU-4800, field emission scanning electron microscope at an accelerating voltage of 3.0 kV and emission current of 10 mA. The fractured surfaces of the materials were sputter-coated with gold before measurement.

### Electrical impedance spectroscopy (EIS)

Electrochemical measurements were performed with a potentiostat/galvanostat (PSTrace4 Palm Sens). The conductivity of each SPE was measured by EIS at an open circuit potential with an applied 10 mV AC potential from 100 kHz to 10 mHz. The thin SPE film including the separator were sandwiched in between blocking electrodes made of stainless steel. The SPEs thin films were first swollen in 2 M Zn(Tf)_2_ (12 μL) for 2 h before assembling the Zn/SPEs/Zn cell. The Zn ion transference number $({\text{t}}_{{{\text{Zn}}^{{2 + }} }}),$ was characterized by chronoamperometry.

### Electrochemical compatibility measurement

The electrochemical compatibility of each SPE film was monitored by the voltage response over long-term zinc charge/discharge cycles on a Neware testing system (Shenzhen Neware CT-4008) at room temperature. The cycling performance of the symmetric Zn/SPEs/Zn cells in a coin cell assembly at a current density of 0.25, 0.75, 1.25, 2.5, 3.75, and 5 mA cm^−2^ was performed for 150 h. The duration of each complete cycle took 25 h. The polarization voltage was recorded and plotted versus time.

## Results and discussion

### Chemical structure of the CMC/PNiPAMx polymer blends

**Figure 1:**
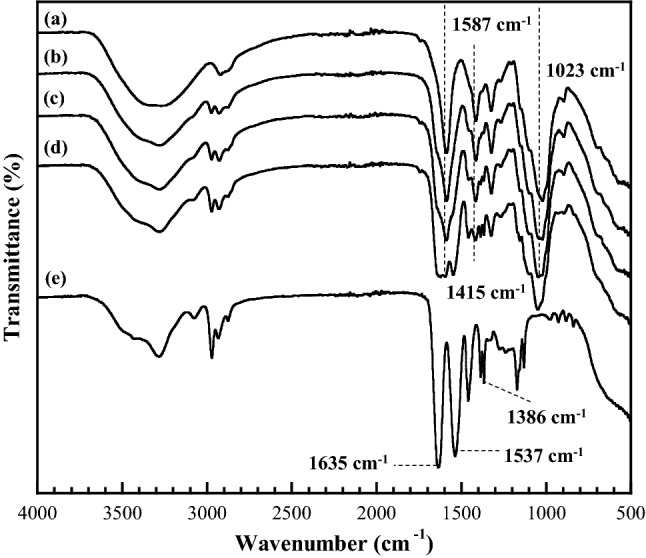
Representative FT-IR spectra of CMC/PNiPAMx polymers with PNiPAM contents of (a) 0 wt% (pure CMC), (b) 10 wt%, (c) 30 wt%, (d) 50 wt% and (e) 100 wt% (pure PNiPAM).

The chemical structure of the CMC/PNiPAMx polymer blends was ascertained by FT-IR spectroscopy since it provides information on the intermolecular interaction between the components of the polymer blend. Moreover, FT-IR analysis is non-destructive and requires only a relatively small amount of sample. Figure 1 showed the functional groups of pure CMC, pure PNiPAM, and the CMC/PNiPAMx films, as determined by FT-IR analysis. The characteristics of CMC functional groups showed a broad band at around 3,369 cm^−1^ of O–H stretching vibration, a peak at approximately 2,920 cm^−1^ due to the C–H stretching, the peak at 1587 cm^−1^ owning to the asymmetric stretching vibration of the carboxylate groups and the peak at 1,415 cm^−1^ of both –CH_2_ scissoring and COO– symmetric stretching vibration. The peak of 1,324 cm^−1^ and 1,023 cm^−1^ were attributed to the O–H bending vibration and the ether groups, respectively. The absorption bands are in good agreement with previous report^23^.

The characteristics of PNiPAM spectrum revealed at the peaks at 3,430 and 3,292 cm^−1^ (secondary amide N–H stretching vibrations), at 2,961 and 2,922 cm^−1^ for the C–H stretching, at 1635 and 1537 cm^−1^ for the amide I (C=O) and amide II (N–H) stretching vibration bands of PNiPAM repeat units. The peak of 1,457 cm^−1^ was attributed to CH_3_ anti-symmetric bending, CH_2_ scissoring, and C–N stretching of amide groups; the peaks at 1,386 and 1,366 cm^−1^ can be assigned to the symmetric bend (or umbrella bends) of the isopropyl groups from PNiPAM.

The spectra of the prepared CMC/PNiPAMx blends (Fig. 1b–d) exhibited all the above characteristic absorption spectral bands of the pure CMC and PNiPAM films, but with some shifts in the band position and changes in the intensity. Therefore, the CMC/PNiPAMx blends had the characteristic features of both CMC and PNiPAM. The overlapped peaks in the O–H stretching vibration in CMC and the secondary amide N–H stretching vibrations in PNiPAM shifted to a lower wavenumber. The peak merging of the carboxylate group in CMC and the amide I group in PNiPAM was observed between wavenumber 1,700–1,500 cm^−1^. Also, the merged vibrational band for CH_2_ scissoring between 1,415 and 1,386 cm^−1^ was investigated for the blend films. The transmission for 1,023 cm^−1^ of the C–O–C group shifted to a lower wavelength and became broader.

These changes suggest a possible specific chemical interaction between CMC and PNiPAM, and indicate the potential compatibility between the two polymers^32^. Generally, CMC structure contains a high electronegative group, which can strongly form the intermolecular hydrogen bonding with the other polymer^33,34^ such as the contained high electronegative nitrogen based PNiPAM with a good proton acceptors characteristic^31^. Therefore, the formation of hydrogen bonds between the functional groups for CMC and PNiPAM polymers can be dominantly proposed. In addition, both CMC and PNiPAM contain hydrophobic and hydrophilic moieties on their chain structure, and hence show amphiphilic characteristics, which provides a high opportunity for H-bond formation. Moreover, the substitution of the CH_2_–COONa group of CMC is heterogeneous in terms of both the substituted position and degree, which increases the complexity of H-bonds^35^.

### Mechanical properties of the CMC/PNiPAMx polymer blends

**Figure 2:**
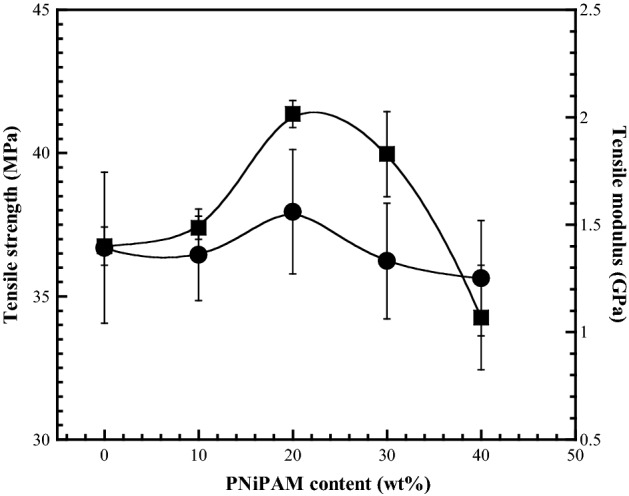
Tensile strength (filled circle) and tensile modulus (filled square) of CMC with different PNiPAM contents. Data are shown as the mean ± 1SD, derived from 5 independent repeats. The curves are drawn as guides to the eyes.

The mechanical properties of a polymer electrolyte are also critical intrinsic factors that influence the safety in use and large-scale manufacture of batteries. The tensile strength and modulus of the CMC/PNiPAMx films are shown in Fig. 2. The CMC film showed a tensile strength and modulus of 35.6 MPa and 1.4 GPa, respectively. By increasing the PNiPAM contents, initially the tensile strength and tensile modulus increased and reached its maximum value 37.9 MPa and 2.1 GPa, respectively, at 20 wt% of PNiPAM content, thereafter they went on decreasing after that. Most CMC/PNiPAMx exhibited better mechanical properties than that of CMC, excepting the CMC/PNiPAM40. However, all CMC/PNiPAMx were far superior to previously evaluate polymer blended CMC films^23,24,35^. Moreover, they are strong enough for cell assembling.

The enhanced mechanical properties of the CMC/PNiPAMx blend films might be attributed to the increased stiffness of the films following the addition of PNiPAM, which contains both –NH and –OH groups that participate in strong intermolecular bonding and electrostatic interactions with the carboxyl and hydroxyl groups of CMC. These interactions may include H-bonds, dipole–dipole, and charge effects. The poor mechanical properties of CMC/PNiPAM40 might be attributed to the excess of low *M*_*w*_ PNiPAM. It could probably act as a plasticizer in the polymer chains, which became flexible with the decrease of the mechanical properties. The excellent mechanical properties ensure that the CMC/PNiPAM can withstand the stress during the cell packaging and act as a good dendrite growth barrier during cell charge/discharge cycling.

### Thermal properties of the CMC/PNiPAMx polymer blends

**Figure 3:**
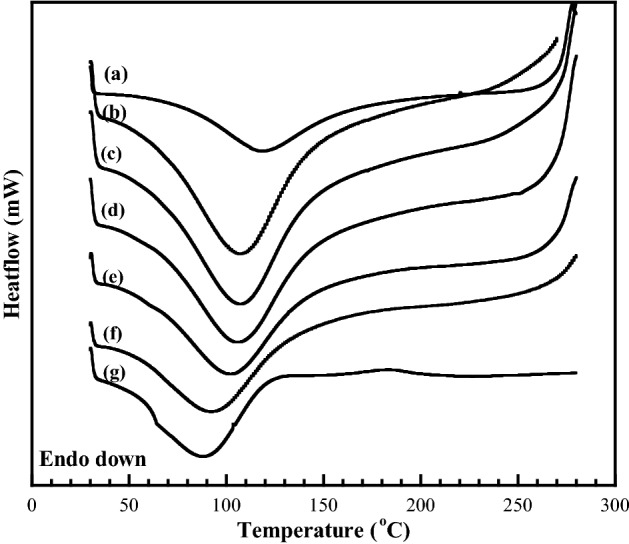
Representative DSC thermograms of CMC with PNiPAM contents of (a) 0 wt% (pure CMC), (b) 10 wt%, (c) 20 wt%, (d) 30 wt%, (e) 40 wt%, (f), 50 wt%, and (g) 100 wt% (pure PNiPAM).

Figure 3 shows the DSC thermograms of the melting region for the different CMC/PNiPAMx blends. A single broad melting peak was observed for all the samples, and the melting temperature (*T*_m_) decreased with increasing PNiPAM contents. The *T*_m_ of CMC was observed at 115 °C, while that of the polymer blends decreased with increasing PNiPAM contents to 90 °C for CMC/PNiPAM50, and the *T*_m_ of pure PNiPAM was 88 °C. That the *T*_m_ of the blend decreased with increasing PNiPAM contents was partly because of a greater suppression of the CMC mobility for cold crystallization and so resulted in less perfect crystallites with enhanced distortions of the intramolecular H-bonds within the CMC crystals. This is similar to that for the reported polymer blends between poly (3-hydroxybutyrate) and cellulose acetate butyrate^36^.

These observations signify the compatibility between the CMC and PNiPAM, which is mainly due to the carboxylate anion and hydroxyl group that facilitated the interaction via H-bond formation. This is an effective benefit on the increment of conductivity when the polymer blends show miscibility without any phase separation between them, whereas the graft polymerization of CMC and PNiPAM showed phase separation and resulted in a negative effect on the ionic conductivity^37^.

**Figure 4:**
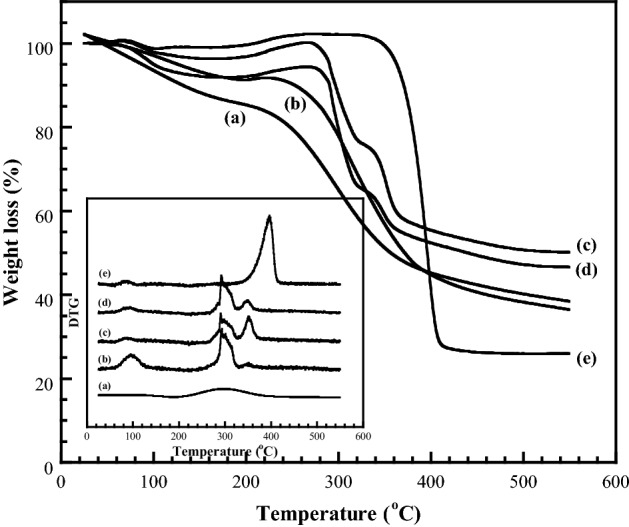
Representative TGA thermograms showing the thermal degradation behavior of CMC with PNiPAM contents of (a) 0 wt% (pure CMC), (b) 10 wt%, (c) 20 wt%, (d) 30 wt%, (e) 40 wt%, and (e) 100 wt% (pure PNiPAM).

The thermal stability of the polymer blends was investigated by TGA, where Fig. 4 shows the TGA data and corresponding derivative thermogravimetry (DTG) curves of the pure CMC, pure PNiPAM, and CMC/PNiPAMx blends under a N_2_ atmosphere and heating at 20 °C min^−1^. The remaining material at 500 °C (carbonaceous residual) from the degradation are reported in Table 1. The pure CMC mainly presented a single thermal degradation process and gradually decomposed as the temperature increased. The initial weight loss of approximately 20% between 25 and 170 °C was due to moisture loss as a result of its hygroscopic nature^38^. Moreover, the nature of CMC absorbs easily moisture from the air, which suddenly decrease the thermal degradation, corresponding with previous studies^26,39,40^.

These behavior was also observed for the pure PNiPAM and polymer blends within the narrow degradation temperature range. The main thermal degradation process, observed over a wide temperature range of 185–420 °C, was the result of the decomposition of the methylcellulose and also from CO_2_ loss following decarboxylation of the COO– functional groups of CMC^34^, and ring scission/dehydration of the CMC structure during the thermal degradation process^41^. The chair of the CMC was observed by the formation of sodium containing species, like Na_2_O and Na_2_CO_3_^42^. Note that PNiPAM is more thermally stable than CMC, as can be seen in Fig. 4e.

The thermal degradation of PNiPAM showed a single-step degradation over the temperature range from 320 to 430 °C. The thermal degradation was initiated by the thermal cleavage of C–N, C–C, and C–O bonds in the polymer chain, and these cleavages gave rise to chain free radicals, which would then become involved in further complicated reaction processes at elevated temperatures, including chain transfer, rearrangement, isomerization, cyclizations, etc., resulting the formation of six- and four-membered amide ring compounds^43,44^.

From the TGA curves of the prepared blends, the degradation process was more complex and took place in two thermal stages. The CMC/PNiPAMx blends were thermally more stable with a much higher onset decomposition temperature compared to the pure CMC (Table 1). This increased thermal stability could be explained by the interaction between the respective functional groups of the CMC and PNiPAM molecules, and might be related to the restricted chain motion in the CMC/PNiPAMx blend that then requires a higher thermal energy to initiate chain scission. These results indicate that the CMC/PNiPAMx blend could be available in the batteries for long term utilization.

### Morphology of the CMC/PNiPAMx polymer blends

**Figure 5:**
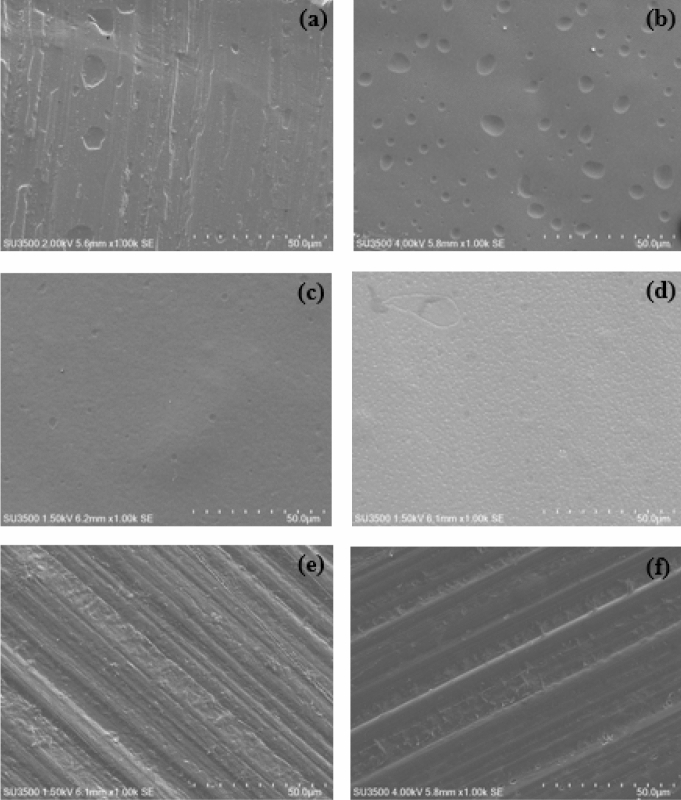
Representative SEM micrographs (× 1,000 magnification) of CMC with PNiPAM contents of (**a**) 0 wt% (pure CMC), (**b**) 100 wt% (pure PNiPAM), (**c**) 10 wt%, (**d**) 20 wt%, (**e**) 40 wt% and (**f**) 40 wt%.

The surface morphology of the CMC/PNiPAMx films was observed using SEM. Figure 5 shows the morphology of the CMC with different PNiPAM contents. The pure CMC displayed a combination of a coarse and smooth surface. The pure PNiPAM showed the smooth surface with the heterogeneous holes whereas that of CMC/PNiPAMx exhibited somewhat different morphologies. A smooth surface with the decrease of pores was obtained with the incorporation of 10 wt% PNiPAM, whereas a smooth and homogenous porous structure was evident at 20 wt% PNiPAM. The smooth surface could be attributed that CMC and PNiPAM used in this composition have a good compatibility and presented more amorphous region in the polymer structure that is suitable to act as a host polymer in the further expansion of the SPEs. The surface morphology then displayed a rough structure at above 20 wt% PNiPAM. The surface roughness were described to the existence of large amounts of crystalline fraction in the CMC/PNiPAMx, which corresponded with the endothermic curves of DSC results. Moreover, there are probably some incompatible parts between polymer components, which would show the individual microstructure of CMC and PNiPAM.

## Electrochemical properties of the CMC/PNiPAMx polymer electrolytes

### Zinc ionic transference $({\text{t}}_{{{\text{Zn}}^{{2 + }} }}),$

**Figure 6:**
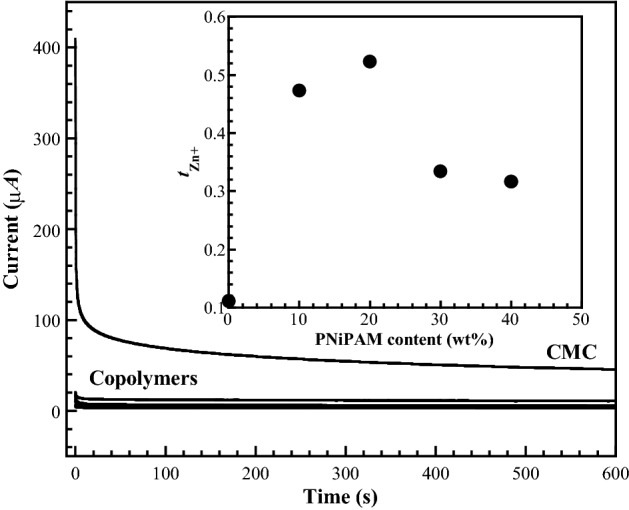
Representative chronoamperometry profiles at room temperature in block cells using Zn metal as both electrodes with a step potential of 10 mV for the prepared CMC/PNiPAMx SPE films. Inset: The ${\text{t}}_{{{\text{Zn}}^{{2 + }} }},$ of SPEs with different PNiPAM contents.

The ${{t}}_{{{\text{Zn}}^{{2 + }} }},$ is an important parameter for lowering the internal resistance and the concentration polarization of metal ions during the charge/discharge cycles of the battery^45^. The ${{t}}_{{{\text{Zn}}^{{2 + }} }},$ can describe the interaction of the proton conduction or differentiate between the current carried by the species of ionic conductor, respectively, even in fully dissociated systems^46^. The plot of the polarized current versus time is shown in Fig. 6, and the ${{t}}_{{{\text{Zn}}^{{2 + }} }},$ was estimated using Eq. (1);

1 $${{t}}_{{{\text{Zn}}^{{2 + }} }} = I_{{\text{s}}} /I_{{\text{o}}} ,$$

where *I*_s_ and *I*_o_ represent the currents at the steady and initial states, respectively.

Figure 6 showed the total current as a function of time. For all SPEs samples, the total current gradually decreased with time at the initial state owning to the ionic species depletion in the electrolyte. The plateau was observed at the fully depleted situation, which the cell is polarized and current flows due to the electron migration across the electrolyte and interfaces at this steady state. The ${{t}}_{{{\text{Zn}}^{{2 + }} }},$ of the CMC-based SPEs with different PNiPAM contents is shown in the inset of Fig. 6. The ${{t}}_{{{\text{Zn}}^{{2 + }} }},$ of the CMC/PNiPAMx blends was larger than that of CMC and showed a maximum value at 20 wt% PNiPAM. It is known that both CMC and PNiPAM contain polyanionic groups in their structure, which can retard the passing through of (C_2_F_6_O_6_S_2_)^2−^ anions. In contrast, the CMC/PNiPAM polyanionic structure favoured the movement of Zn^2+^ ions, which corresponds with the porous SPEs in a Li battery system^26^. The highest ${{t}}_{{{\text{Zn}}^{{2 + }} }},$ of 0.54 was found with the CMC/PNiPAM20 blend, due to the porous structure of the host polymer, as shown in the SEM analysis, which is a significant factor in ion transference.

### Ionic conductivity

**Figure 7:**
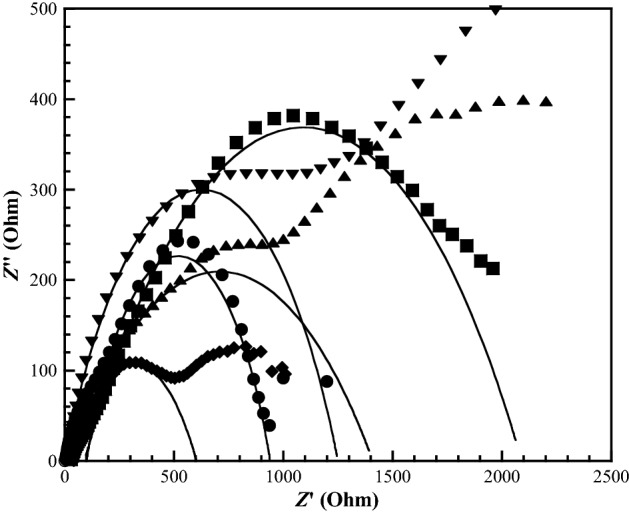
Representative Nyquist plots of the symmetrical cell for the CMC/PNiPAMx SPE systems with PNiPAM contents of (filled circle) 0 wt% (pure CMC), (filled square) 10 wt%, (filled diamond) 20 wt%, (filled triangle) 30 wt%, and (filled inverted triangle) 40 wt%.

Ionic conductivity is generally regarded as a crucial parameter of SPEs for energy storage applications, and provides information on the total transport of charges. The ionic conductivity (δ) of SPEs can be calculated from Eq. (2);

2 $$\delta = \frac{1}{{{\text{R}}_{{\text{b}}} }}\frac{{\text{d}}}{{\text{s}}},$$

where *d* is the thickness of the SPEs thin film, *S* is the area of electrodes contained within the SPE film, and *R*_b_ is the bulk resistance. From Eq. (2), the ionic conductivity of the electrolytes was calculated from the SPE film resistance, as determined from the complex impedance spectra or the Nyquist impedance plot (Fig. 7). To analyse the characteristics of the Nyquist impedance plot, Z view software was used to fit the spectrum by using an equivalent circuit, in which the electrode resistance is in series with the parallel combination of the electrolyte resistance and capacitance. This approach is often used because it is simple, fast, and can provide a complete picture of the system^22,47^. The electrolyte resistance is determined from the intercept of the spur extrapolated to the real axis.

Figure 7 shows the Nyquist impedance plots for the assembled Zn/SPEs/Zn cell structures. The spectra show two different parts: an incomplete semicircle in the high frequency and a spur in the lower frequency. The semi-circle part of the impedance spectrum in the high frequency region is related to the ionic conduction in the bulk of the sample and is due to the parallel combination of the bulk resistance (due to migration of protons) and bulk capacitance (due to immobile polymer chains)^48^. The obtained values of the ionic conductivity are reported in Table 1. The ionic conductivity of the polymer electrolyte films obtained at room temperature were in the range of 0.56–1.68 × 10^–4^ S cm^−1^, with the highest value obtained at a PNiPAM content of 20 wt%. The increment in the ionic conductivity can be attributed to the ion association and the migration of the ions through the porous structure of CMC/PNiPAM20.

For CMC/PNiPAM20, there was probably a large amount of effective charge carriers in the chain, which could interact with the zinc ions. Hence, there will be more sites at which ion hopping and exchange can take place leading to the highest ionic conductivity. In addition, it is well known that ion transport, principally occurs in the amorphous regions. It seems that the CMC/PNiPAMx exhibited more crystalline than the pure CMC as revealed in the increase of the area under the endothermic of DSC thermograme, resulting the low ionic conductivity. Moreover, the ionic conductivity of SPEs depends not only on the amorphous content, but also the amount of electrolyte uptake, the interfacial contact with the electrodes, as well as the chemical structure characteristics of the SPEs films^49^. At room temperature, all CMC/PNiPAMx presented the transparent films (not show all). When the CMC/PNiPAMx were swollen with the electrolyte aqueous solution and the external stimuli such as temperature, ionic field and electrochemical response were applied. CMC remained a transparent SPE. The CMC/PNiPAM20 exhibited a semi-transparent SPE due to the uniform bonding of hydrophilic PNiPAM (amide groups) with CMC and water through hydrogen bonds. The electrolyte solution could be embedded in the hydrophilic region of swollen CMC/PNiPAMx to function as a conductive path and cause a decreased the bulk resistance, rendering high ionic conductivity. Whereas the SPEs films became the opacity with PNiPAM above 20 wt% due to changes in PNIPAm conformation from hydrophilic to hydrophobic as shown in Supplementary Fig. S1. The hydrophilic amide groups will be shelled by the hydrophobic isopropyl-methyl groups because the PNIPAM could form intra and intermolecular interactions with itself, attributed to the formation of dehydrated PNiPAM regions, the CMC/PNiPAMx swollen films are expected to have dehydrated PNiPAM region and CMC region which resulted in the opacity. The zinc ions would be almost entrapped in the hydrophobic shrinkage of PNiPAM, which blocked conductive pathways and lost the ability to transport across a long distance, leading to higher the bulk resistance and low ionic conductivity^50,51^.

We attempted to increase the ionic conductivity by the incorporation of various Zn(Tf)_2_ contents into the CMC/PNiPAM20 matrix. However, the results (Supplementary Fig. S2) revealed a decline in the ionic conductivity with increasing Zn(Tf)_2_ contents which could be attributed to the aggregation of the overcrowded ions, which then reduces the charge carriers process, and the re-association of the ions into neutral aggregates^52^, where the excess salt recrystallized out of the polymer, and so reduced the conductivity of the samples. Low Mw aprotic plasticizers or/and dopants have previously been incorporated in CMC host polymers to achieve a high ionic conductivity up to 10^−4^ S cm^−1^. However, the obtained SPEs showed a weak mechanical strength or were without any report of the mechanical property^22,47,53,54^, and this could adversely affect the safety in use and large-scale manufacture of the batteries.

### Electrochemical compatibility

**Figure 8:**
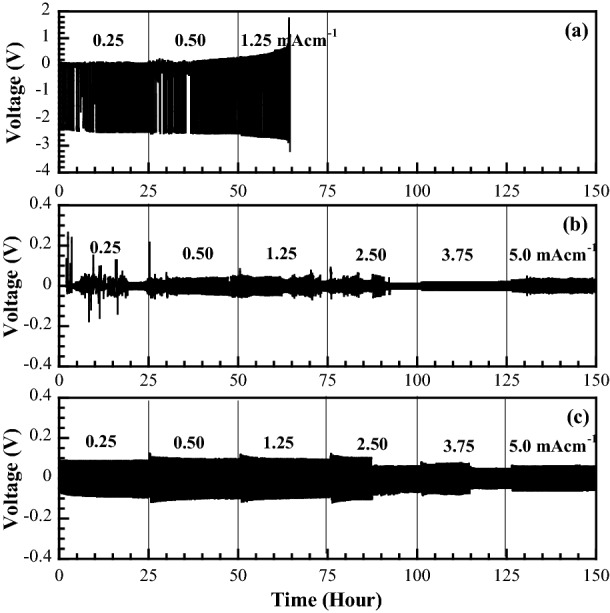
Half Zn battery cell testing for charge/discharge scycle of (**a**) CMC, (**b**) CMC/PNiPAM20, and (**c**) 15 wt% Zn(Tf)_2_ containing CMC/PNiPAM20.

The typical charge/discharge cycling performance is shown in Fig. 8, comparing CMC, CMC/PNiPAM20, and 15 wt% Zn(Tf)_2_ containing CMC/PNiPAM20 with the current density from 0.25 to 5 mA cm^−2^. For a CMC-based electrolyte, the over-potential for the charge/discharge cycle of zinc significantly increased with time and current density. Short-circuiting occurred during the cycling process after 60 h, indicating that zinc dendrites had penetrated the CMC film^55^. Whereas, both CMC/PNiPAM20 and CMC/PNiPAM20-Zn(Tf)_2_ complex samples remained stable in the charge/discharge cycling for a total of 150 h without any potential damage. The absence of any short-circuit phenomenon observed during the 150 h polarization for the cyclic current densities indicates the good compatibility between the polymer and zinc electrode and so the safety in the development of zinc ion batteries. The fluctuation in the voltage signal from the former sample may also be a consequence of the hydrophilic/hydrophobic nature transition of PNiPAM due to the applied current. Therefore, in future we aim to increase the ionic conductivity by increasing the porosity of the SPEs for the CMC/PNiPAM20 zinc electrolyte complex.

## Conclusions

The high ion conductivity with the high safety SPEs based on a CMC/PNiPAM20 blended Zn ion battery was developed in this research. The addition of PNiPAM to CMC resulted in enhanced mechanical and thermal properties. The incorporation of 20 wt% PNiPAM (CMC/PNiPAM20) showed the greatest tensile strength and modulus among the CMC/PNiPAMx blends. Moreover, CMC/PNiPAM20 had a porous structure, which supported the movement of Zn^2+^ in the SPEs and resulted in a high Zn^2+^ ion transference number and ionic conductivity. In addition, the PNiPAM stabilized the cyclic performance by suppressing dendrite formation, which otherwise causes a short circuit in the battery cell. From the results, the developed CMC/PNiPAM20 based SPE is a promising material for high ionic conductivity and stability in Zn ion batteries.

## Supplementary information

Supplementary information.
