# Supplementary material for: Properties enhancement of carboxymethyl cellulose with thermo-responsive polymer as solid polymer electrolyte for zinc ion battery
Source: Sci Rep. 2020 Jul 28;10:12587. doi: 10.1038/s41598-020-69521-x (PMC7387535; doi:10.1038/s41598-020-69521-x)
Supplement: Supplementary file 1 — Supplementary information. [file 41598_2020_69521_MOESM1_ESM.docx]

**Supplementary Information**

**Properties enhancement of carboxymethyl cellulose with thermo-responsive polymer as solid polymer electrolyte for zinc ion battery**

Isala Dueramae^1^, Manunya Okhawilai^1,2,*^, Pornnapa Kasemsiri^3^, Hiroshi Uyama^4^, Rio Kita^5^

^1^Metallurgy and Materials Science Research Institute, Chulalongkorn University, Bangkok 10330, Thailand.

^2^Center of Excellence in Responsive Wearable Materials, Chulalongkorn University, Bangkok 10330, Thailand.

^3^Sustainable Infrastructure Research and Development Center and Department of Chemical Engineering, Faculty of Engineering, Khon Kaen University, Khon Kaen 40002, Thailand.

^4^Department of Applied Chemistry, Graduate School of Engineering, Osaka University, Osaka 565-0871, Japan.

^5^Department of Physics, Tokai University, Kanagawa 259-1292, Japan.

^*^Corresponding author email: [Manunya.o@chula.ac.th](mailto:Manunya.o@chula.ac.th)


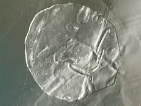

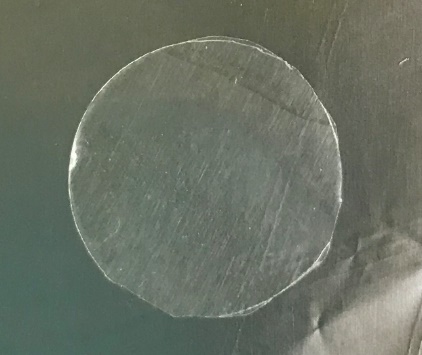


**(b)**

**(a)**

**(c)**


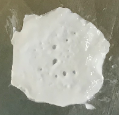

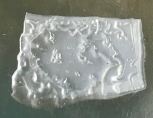


**(d)**

**Figure S1.** The physical appearance of films (a) without and (b-d) with the electrolyte solution and the external stimuli for CMC with various PNiPAM contents of (b) 0wt% (pure CMC), (c) 20wt%, and (d) 40wt%.

**Figure S2.** Ionic conductivity of CMC/PNiPAM20 with various amount of Zn(Tf)_2_ at room temperature. Curves are drawn to guide eyes.
